# Contributions of neighborhood violent crime and perceived neighborhood safety to cognition and mental health in the adolescent brain cognitive development study

**DOI:** 10.1016/j.dcn.2025.101660

**Published:** 2025-12-18

**Authors:** Patrick M. Lindsley, Nourhan M. Elsayed, Deanna M. Barch

**Affiliations:** a Department of Psychological and Brain Sciences, Washington University in St. Louis, St. Louis, MO 63105, USA; b Department of Psychiatry, Washington University School of Medicine, St. Louis, MO 63110, USA; c Department of Radiology, Mallinckrodt Institute of Radiology, Washington University School of Medicine, St. Louis, MO 63110, USA

**Keywords:** Neighborhood violence, Neighborhood safety, Mental health, Cognition, Developmental adversity

## Abstract

**Introduction:**

This study investigates how objective neighborhood violence and perceived neighborhood safety (PNS) relate to adolescent mental health, cognitive performance, and brain structure. We examined whether PNS moderated the effects of neighborhood violence, explored neural correlates of PNS, tested longitudinal relationships, and assessed sociodemographic and psychological predictors of PNS.

**Methods:**

Data from the ABCD Study (n = 11,865) were used to examine associations between PNS, violent crime, and youth outcomes. Measures included youth and caregiver surveys, FBI crime data, NIH Toolbox cognitive tasks, and MRI-based brain volume metrics in stress-related regions.

**Results:**

PNS, but not objective violence, was associated with fewer mental health symptoms and better cognitive performance. PNS was also related to sociodemographic variables and greater right amygdala volume. Longitudinally, baseline PNS predicted later cognitive performance, while baseline mental health and working memory predicted future PNS, indicating bidirectional effects.

**Discussion:**

Perceived safety, rather than objective crime, was linked to adolescent mental health, cognition, and brain structure. PNS was influenced by sociodemographic and psychological factors, and mental health predicted declines in safety perception. These findings emphasize the developmental importance of subjective environmental experiences.

## Highlights

• Perceived neighborhood safety was linked to better mental health and cognition.
• Objective neighborhood violence was not associated with these outcomes.
• Greater perceived safety was related to larger right amygdala volume.
• Findings suggest mental health symptoms may influence how youth perceive their neighborhoods.

## 1 Introduction

Violent crime rates have been steadily declining in the United States since the 1990s (Statista, 2022). However, public perception tells a different story—over the past decade, anywhere from 60 % to 80 % of Americans have reported believing that crime is worsening each year (Gallup, 2020). This disconnect suggests that perceptions of crime are shaped by more than crime rates themselves. A growing body of research indicates that subjective experiences of threat, rather than objective measures alone, are more strongly associated with mental health outcomes (Danese and Widom, 2020, Goldman-Mellor et al., 2016). As such, understanding how subjective and objective indicators of neighborhood safety interact may help clarify their respective roles in mental health and cognitive functioning. The present study aimed to clarify the relationship between neighborhood violence, perceived neighborhood safety, and mental health and cognitive outcomes. By examining both cognitive and mental health outcomes, this study provides a comprehensive view of how perceived neighborhood safety relates to a wide array of outcomes, aiming to further characterize the correlates of this neighborhood-level measure of adversity.

Higher levels of objective measures of neighborhood violence, such as police-reported crime rates or geocoded crime data, have been linked to adverse psychological and cognitive outcomes, particularly among youth (Baranyi et al., 2021). Exposure to community violence is associated with increased risks of internalizing and externalizing disorders, including post-traumatic stress disorder (PTSD), aggression problems, and depression (Cooley-Quille et al., 2001; P. J. Fowler et al., 2009; Gorman-Smith and Tolan, 1998). Complementing these findings, McCoy et al. (2016) found that pre‑adolescents living in high‑crime neighborhoods showed greater selective attention to negative emotional stimuli and differences in emotion appraisal and regulation, suggesting that neighborhood crime may shape cognitive processes—such as attention and interpretation—that underlie emotional responding. Chronic exposure to violent environments can activate biological stress systems, such as the hypothalamic-pituitary-adrenal (HPA) axis, leading to heightened cortisol reactivity and potential alterations in neural development (McEwen, 2012, McLaughlin et al., 2014). These stress-related changes may disrupt functioning in brain regions responsible for cognitive control and emotion regulation, including the prefrontal cortex and amygdala, potentially contributing to altered and impulse control (Blair and Raver, 2012, Sharkey et al., 2012). The consequences of objective violence are likely cumulative and developmentally sensitive, with early exposure exerting more pronounced effects on neural plasticity and long-term functioning (Margolin and Gordis, 2000, McLaughlin et al., 2014). Despite these documented associations, the relationship between objective violence and psychological or cognitive outcomes likely varies depending on individual, familial, and contextual factors, underscoring the need to examine how perceptions of safety may shape or buffer these effects.

Perceived neighborhood safety (PNS), typically measured by self-reported feelings of safety within a twenty-minute walk from home, has been widely linked to mental health well-being (Aneshensel and Sucoff, 1996, Wang et al., 2019). Higher PNS is associated with better overall mental health (Wang et al., 2019) and lower levels of externalizing symptoms, emotional disorders, and general mental health disturbances (Aneshensel and Sucoff, 1996, Meltzer et al., 2007, Pettit et al., 1999). PNS has also been linked to cognitive functioning, with greater perceived safety predicting better working memory, executive function, and reading comprehension (Assari et al., 2021, Conley et al., 2022, Martin-Storey and Crosnoe, 2014). Traditionally, perceived neighborhood safety was assumed to differ as a function of crime rates, but recent findings challenge this assumption (Austin et al., 2002, Velasquez et al., 2021). Socioeconomic factors, such as neighborhood-level and family-level socioeconomic disadvantage, as well as other community-level characteristics like social cohesion, appear to be stronger predictors of PNS than actual crime statistics (Choi and Matz-Costa, 2018, De Jesus et al., 2010, Henry et al., 2019). Conversely, factors like pre-existing health concerns and greater psychopathology have been linked to lower PNS (Sun et al., 2012), further underscoring that perceptions of safety are likely influenced by environmental and psychological contexts.

A meta-analysis by Baranyi et al. (202e2w11) found that PNS (r = 0.08 for depression; r = 0.07 for psychological distress) showed stronger associations with mental health outcomes than those examining objective neighborhood violence (r = 0.02 for depression; r = 0.03 for psychological distress). This pattern of findings raises a critical question: if perception of threat may be more strongly associated with mental health rather than objective measures of threat, could it also serve as a protective factor? Some studies suggest that a strong sense of safety may buffer against the psychological effects of neighborhood violence, mitigating stress responses and reducing vulnerability to mental health disorders (Choi and Matz-Costa, 2018, Wilson-Genderson and Pruchno, 2013). However, the extent to which PNS moderates the relationship between objective neighborhood violence and psychological or cognitive outcomes remains unclear. Additionally, the neural mechanisms underlying these relationships are not well understood.

Higher levels of neighborhood disadvantage have been associated with reduced volumes in the amygdala, hippocampus, and prefrontal cortex (Hanson et al., 2023, Hunt et al., 2020, Taylor et al., 2020). Conversely, larger brain structures and greater gray matter are generally associated with increased resilience to the risks posed by neighborhood disadvantage (Suarez et al., 2025). Variation in these stress and emotion regulation-related brain areas– particularly the amygdala–many help explain how perceptions of neighborhood safety relate to mental health and cognition, though findings to date are mixed. Several studies have examined amygdala activity in relation to lower perceived neighborhood safety, with some reporting increased activity in the left amygdala (Ortiz-Whittingham et al., 2024), others in the right (Suarez et al., 2022), and some finding no altered activity at all (Cai et al., 2024). Decreased amygdala volume has been associated with early-life adversity (Vannucci et al., 2023), potentially reflecting excitotoxic effects of adversity-related stress (Hanson and Nacewicz, 2021). To our knowledge, no studies have identified associations between self-reported PNS and structure or activity in the prefrontal cortex or the hippocampus. Given the well-established roles of these regions in stress and threat processing (Alexandra Kredlow et al., 2022, Grupe et al., 2019, Roberts, 2020, Tanriverdi et al., 2022), along with findings linking them to other neighborhood-level stressors (Taylor et al., 2020, Vargas et al., 2020), it is plausible they may also be involved in neighborhood safety perceptions. Further, given links between PNS and elevated cortisol (Stephens et al., 2021, Thayer, 2017), the evidence for the neurotoxic effects of prolonged cortisol exposure on the amygdala, prefrontal cortex, and hippocampus (Chen and Baram, 2016, Joos et al., 2019), and the hypothesis that these neurotoxic effects contribute to volumetric changes (McEwen, 1999, White et al., 2023), examining the volumes of these regions is necessary to understand potential neurobiological mechanisms.

The present study examines how both objective neighborhood violence and perceived neighborhood safety (PNS) relate to youth mental health, cognitive functioning, and brain structure. These domains are interrelated: chronic perceptions of threat may alter stress-regulatory neural systems, which in turn influence emotional regulation and attention (Blair and Raver, 2012, Tottenham and Galván, 2016, Zsido and Kiss, 2024). We included brain structure to test whether neural differences—particularly in regions sensitive to stress exposure such as the amygdala, hippocampus, and prefrontal cortex—might represent a biological pathway through which PNS relates to mental health and cognition (Hanson et al., 2023, Kawamoto et al., 2023, Roth et al., 2018). Thus, incorporating brain data allows us to assess not only whether PNS correlates with behavioral outcomes, but also whether it may be related to differences in neural architecture that supports those behaviors.

First, the study examined whether individual and contextual factors–such as child-level psychopathology, group-identity status (i.e., race, ethnicity, sex, gender, sexual orientation), and markers of socioeconomic disadvantage (e.g., lower income, higher neighborhood-level poverty and unemployment, lower educational attainment–were associated with adolescents’ perceptions of neighborhood safety, independent of exposure to objective neighborhood violence. It was hypothesized that youth with greater psychopathology and greater socioeconomic disadvantage, as well as those identifying with minoritized social groups, would report lower PNS. Second, the associations between PNS and objective neighborhood violence with mental health and cognitive outcomes were examined, hypothesizing that greater PNS and lower levels of objective neighborhood violence would be associated with fewer mental health symptoms and better cognitive task performance.

By incorporating both subjective (PNS) and objective (i.e., crime statistics) measures of neighborhood safety, this study also examined whether perceptions of safety moderate the effect of objective indicators of neighborhood violence on psychological and cognitive functioning. Specifically, we investigated whether greater PNS would weaken the association between objective neighborhood violence and cognition and mental health. This hypothesis is consistent with findings that subjective experience and perceptions may buffer the biological and psychological impact of neighborhood condition (Cutrona et al., 2000, Weden et al., 2008), consistent with the idea that perception can shape the relationship between objective experience and associated outcomes (Danese and Widom, 2020). We also examined the neural correlates of PNS, focusing on regions consistently linked to chronic stress exposure and emotional regulation, including the amygdala, hippocampus, and prefrontal cortex. Prior work indicates that adversity and perceived threat are often associated with reduced amygdala and hippocampal volume (Foell et al., 2019, Grupe et al., 2019, Picci et al., 2022). Accordingly, we hypothesized that lower PNS (i.e., greater perceived unsafety) would relate to smaller amygdala, hippocampal, and prefrontal volumes, consistent with models suggesting that sustained threat perception may relate to variation in stress-sensitive neural systems (Başgöze et al., 2021, O’Donovan et al., 2013). Lastly, using longitudinal data, this study preliminarily investigated the directionality of these relationships–examining whether perceived safety predicts future mental health and cognition, or whether preexisting mental health symptoms might shape future perceptions of safety. This is a pre-registered project. A summary of pre-registration deviations can be found in the supplemental section.

## 2 Methods and materials

### 2.1 Participants

Data from the 5.0 data release of the Adolescent Brain Cognitive Development (ABCD) study was used for the analyses. The multi-site study includes 11,865 adolescents (and their guardians) recruited at baseline (age 9–10) at 21 research sites across the United States. Recruitment primarily took place in local elementary schools, through community outreach, referrals, and with the use of state twin-registries, for sites that specialize in twin children. More descriptive information on ABCD recruitment practices have previously been published (Garavan et al., 2018).The ABCD study aimed to explore factors associated with healthy physical and mental health in children. Participants were primarily recruited through schools in specific catchment areas across 21 ABCD Study sites, using a multistage probability sampling method to ensure sociodemographic diversity. Due to the intensive and longitudinal nature of the data collection, some participants had missing data on specific variables. For each analysis, participants with missing data on any variables included in the model were excluded using listwise deletion, resulting in analysis-specific sample sizes Data was used from the baseline (age 9–10) and year two (age 11–12) timepoints. While ABCD data are available at additional follow-ups, PNS, cognition, and structural MRI were only currently available at baseline and year two.

## 3 Measures

### 3.1 Demographic Information

At baseline, parents provided demographic and personal information about their child through interviews and online surveys. For these analyses, we used data on the child’s sex, race, sexual orientation, and age. Family income was also reported at this time. Additionally, participants’ home addresses were used to calculate the Area Deprivation Index (ADI; Kind et al., 2014; Singh, 2003), which reflects the socioeconomic disadvantage of their neighborhood, and their census tract population density. We winsorized census tract population density data, due to a cluster of extreme outliers that appeared to be driven by geographical and urban design factors, including proximity to airports, public parks, and industrial zones. The ADI is a composite index derived through factor from 9 of the original 17 indicators, including variables such as percentage of families living in poverty, unemployment rates, and neighborhood level of percentage of educational attainment. For the present analyses, the ADI was reverse coded, such that greater ADI values reflected lower neighborhood disadvantage. More information about the specifics of the factor analysis used to derive this iteration of ADI can be found in Taylor et al. (2020).

### 3.2 Perceived neighborhood safety

Participants completed the Neighborhood Safety and Crime questionnaire, a one question, youth-report measure asking the participant to rate their agreement with the statement “My neighborhood is safe from crime” on a Likert rated scale (Range = 1–5) (Mujahid et al., 2007; PhenX Toolkit: Protocols, n.d.). Assessments from this measure at baseline (age 9–10) and year two (age 11–12) were used in analyses.

### 3.3 Objective neighborhood violence

Objective Violent Crime data was obtained from the Uniform Crime Reporting (UCR) Data, which is part of the ABCD study’s Linked External Data (LED). County-wide violent crime prevalence was measured based on participants' baseline residential information (age 9–10) (Investigation, 2014). Crime rates were referenced from the 2010 UCR crime reports, corresponding to the counties where participants lived at baseline.

### 3.4 Mental health

Caregiver-reported Child Behavior Checklist (CBCL) DSM-oriented subscales were used to index behavioral and emotional functioning at baseline and year two (Achenbach, 2009). The CBCL is a widely validated measure of psychopathology in children and adolescents, with DSM-oriented scales mapping to psychiatric symptoms (Achenbach, 2009, Barch et al., 2018). The DSM-oriented scales included depressive problems, anxiety problems, somatic problems, attention deficit/hyperactivity (ADHD) problems, oppositional defiant problems, and conduct problems (Nakamura et al., 2009). Summed scores from these subscales were calculated to capture overall symptom burden in each domain. For the first analysis, Child-level psychopathology was created by averaging CBCL sub-scale scores at baseline.

### 3.5 Cognition

Cognitive function was assessed using tasks from the NIH Toolbox Cognitive Battery, a validated set of computerized cognitive assessments designed for large-scale studies (Weintraub et al., 2013). Data from both baseline and year two were used in analyses. Receptive language was measured using the NIH Toolbox Picture Vocabulary Task (Gershon et al., 2014). The NIH Toolbox Flanker Inhibitory Control Task was used as a measure of attentional control and response inhibition. Working memory was evaluated with the NIH Toolbox List Sorting Task, which requires participants to sequence and recall information. Cognitive flexibility and attention were measured using the NIH Toolbox Dimensional Change Card Sort Task. Processing speed was assessed with the NIH Toolbox Pattern Comparison Task (Carlozzi et al., 2015). Episodic memory was measured using the Toolbox Picture Sequence Memory Task (Bauer et al., 2013). Reading decoding skill was evaluated using the NIH Toolbox Oral Reading Recognition Task. All tasks were administered at baseline, but List Sorting and Dimensional Card Change were not administered at year two.

### 3.6 Brain area volumes

Baseline structural MRI data were collected using 3 T MRI scanners (Siemens, Philips, and General Electric) with standardized imaging protocols across platforms. A 3D T1-weighted structural scan (1-mm voxel resolution) was acquired while participants viewed a child-appropriate movie. Motion detection and correction software were applied in real time at Siemens and GE sites to minimize movement artifacts.

Imaging data were processed using FreeSurfer version 5.3.0, following established quality control protocols (Fischl, 2012, Hagler et al., 2019). Brain regions of interest were selected based on relevance to stress responses, including the superior prefrontal cortex (superior frontal gyri and superior frontal sulci), medial prefrontal cortex (medial frontal gyri and medial frontal sulci), inferior prefrontal cortex (inferior frontal opercular part and inferior frontal triangular part), left hippocampus, right hippocampus, left amygdala, and right amygdala. Cortical Regions were defined according to the Destrieux atlas (Destrieux et al., 2010), and subcortical volumes were obtained from FreeSurfer’s automatic segmentation (aseg) output. Quality control was performed by the ABCD consortium’s image processing team, assessing for image artifacts or reconstruction inaccuracies. Imaging data for each individual was either recommended or not recommended for use based off their quality assessment (Hagler et al., 2019). To ensure data integrity, scans rated as not recommended for use were excluded (Dai et al., 2022, Parsons et al., 2024).

### 3.7 Behavioral inhbition systems (BIS) scores and direct violence exposure

To determine whether perceptions of neighborhood safety were primarily reflective of broader negative affect or traits associated with anxiety proneness instead of a measure of perceptions of safety, additional analyses were conducted using Behavioral Inhibition (BIS) scores (as a potential indicator of neuroticism). Given prior literature demonstrating the role that neuroticism and negative affect play in threat perception (Lommen et al., 2010, Tamir et al., 2006), determining whether PNS is related to mental health or cognitive function independent of neuroticism would help address the construct validity of PNS reports. The BIS data were derived from baseline and year 2 of the study. BIS sum scores were z-scored and used for analyses as a proxy for possible neuroticism (Carver and White, 1994). For this scale, larger values indicated greater behavioral inhibition. We also conducted follow-up analyses using self-report data on direct violence exposure, derived from the PhenX LToolkit Life Events Inventory (PhenX Toolkit: Protocols, n.d.). From this inventory, we used two variables, one asking if the participant had observed a violent crime and the other asking if a participant had been the victim of a violent crime. These items were summed to create a composite index of direct violent crime exposure (range = 0–2), with high scores indicating greater exposure.

#### 3.7.1 Analysis

All analyses were conducted using linear mixed-effects models to account for the nested structure of the data, with random intercepts for family and site. Models were implemented using R and the lme4 package. To examine factors associated with perceived neighborhood safety (PNS), we tested a model that included objective neighborhood violence, area deprivation index (ADI), combined family income, caregiver-reported community cohesion, child psychopathology (CBCL DSM-oriented subscales), and census tract-reported population with PNS as the dependent variable. Data from both baseline and year two were analyzed together using a hierarchical linear model (HLM), with observations nested within participants to account for the repeated measures across time.

For each set of analyses described below, Bonferroni correction was used across the mental health models and across the cognition models separately. To assess the relationship between neighborhood factors and youth outcomes, we used linear mixed-effects models to predict caregiver-reported mental health symptoms and performance on the NIH Toolbox cognitive tasks. Thirteen separate models were run for each mental health subscale (depression, anxiety, somatic symptoms, ADHD, oppositional defiant disorder, conduct disorder) and each cognitive task (Flanker, List Sorting, Pattern Comparison, Picture Sequence, Card Sort, Picture Vocab, Oral Reading). Models included objective neighborhood violence and PNS as primary predictors and controlled for interview age, sex, site, family income, area deprivation, and population density. We corrected based off of six models for the mental health analyses and seven models for the cognition models. Interaction models were used to test whether PNS moderated the association between neighborhood violence and youth outcomes. Each model included an interaction term between PNS and objective neighborhood violence, along with the same covariates as in the main effects models. Analyses were conducted using a HLM format, with both baseline and year two data, and observations were nested within participant. Corrections were done based off of six models for the mental health analyses and seven models for the cognition analyses. In the review process, we received suggestions to examine the potential role of personal experiences with violence and to examine whether PNS might be capturing personality or temperament. Thus, we re-ran the previously described analyses, involving PNS and mental health and cognition, twice—once including behavioral inhibition (BIS) as a covariate and again including direct violence exposure as a covariate. All other covariates (objective neighborhood violence, sex, age, family income, area deprivation, population density) were retained and models were nested within participant. Additionally, we examined the relationship between PNS and BIS scores, using a linear mixed effects model that included BIS scores as a dependent variable and PNS as an independent variable, controlling for age, sex, objective neighborhood violence, area deprivation index, family income, and population density. This model was nested within participant. Two other linear mixed effects models were used, one including baseline BIS as an independent variable and year 2 PNS as a dependent variable, controlling for baseline PNS. The other included baseline PNS as a dependent variable and year 2 BIS as a dependent variable, controlling for baseline BIS. For both of these models, all other covariates (objective neighborhood violence, sex, age, family income, area deprivation, population density) were retained and models were nested within research site and family. Bonferroni adjustment was applied for the longitudinal models that included BIS and PNS, correcting based off of two models.

To examine neural correlates of PNS, we conducted four linear mixed-effects models with volume of the right amygdala, left amygdala, left hippocampus, right hippocampus, superior prefrontal cortex, medial prefrontal cortex, and inferior prefrontal cortex as the dependent variables. PNS was the primary predictor. Models controlled for intracranial volume (ICV), sex, age, site, family income, population density, ADI, and county-wide crime prevalence. Bonferroni correction was applied across seven four models, correcting for seven models. We then tested whether brain regions associated with PNS–specifically, the right amygdala–were also associated with youth mental health or cognitive outcomes. These models included right amygdala volume as the predictor and each mental health and cognitive outcome as dependent variables. Covariates were consistent with the brain models above and the same Bonferroni correction approach was used (i.e., corrected for six mental health models and seven cognitive models). A HLM format was used to conduct analyses on both baseline and year two data, nesting observations within participant.

For longitudinal analyses, we ran two sets of linear mixed-effects models to explore potential directionality between PNS and youth outcomes. In the first set, baseline PNS was used to predict year two mental health and cognitive outcomes, controlling for baseline levels of those outcomes. Bonferroni correction was conducted based off six mental health models and three cognitive models. In the second set, baseline mental health and cognitive outcomes were used to predict year two PNS, controlling for baseline PNS. Bonferroni correction was applied, correcting based off six models for mental health and five for cognition.

## 4 Results

### 4.1 Demographics

The sample included slightly more males than females, with 45.6 % of the participants being female. Most children were born full-term, with over 92 % born at 36 weeks of gestation or later, and their average birth weight was approximately seven pounds. The cohort was diverse, including 2.1 % Asian, 11.9 % Black, 19.3 % Hispanic, 9.9 % identifying as Other, and 56.9 % White.

### 4.2 Factors related to perceived neighborhood safety beyond neighborhood violence

A linear mixed-effects model was used to examine factors beyond neighborhood violence that may be associated with perceived neighborhood safety (PNS). Results are shown in Table 1. Objective measures of neighborhood violence were not associated with PNS. Greater perceptions of neighborhood safety were associated with lower area deprivation (i.e., greater neighborhood resources), higher combined family income, and stronger sense of community cohesion. Conversely, higher levels of child psychopathology and living in areas with greater population density were associated with lower perceptions of neighborhood safety.

**Table 1:** Associations Between Sociocultural and Identity Factors and Perceived Neighborhood Safety, Beyond Objective Neighborhood Violence.

	Estimate(β)	SE	t value	p
Race (Effect coded)	-0.001	0.027	−0.027	0.978
Objective Neighborhood Violence	-0.015	0.021	−0.724	0.474
Census Tract Population Density	-0.079	0.013	−5.899	< .001 *
Sex assigned at birth (Effect coded)	-0.027	0.019	−1.461	0.144
Age	-0.030	0.018	−1.712	0.087
Area Deprivation Index (Reverse Coded)	0.213	0.014	15.387	< .001 *
Combined Family Income	0.024	0.005	4.462	< .001 *
Sexual Orientation	-0.032	0.021	−1.522	0.128
Child Level Psychopathology	-0.089	0.013	−6.721	< .001 *
Community Cohesion	0.008	0.001	8.072	< .001 *

All variables, excluding race and sex assigned at birth, were scaled and centered. Race was effect coded, such that white participants were coded as −.5 and non-white participants were coded as.5. Sex assigned at birth was also effect coded, such that male participants were coded as −.5 and female participants were coded as.5. * indicates significance (p < .05).

### 4.3 Relationship of exposure to objective neighborhood violence and perceived neighborhood safety to cognition and mental health outcomes

**Fig. 1:**
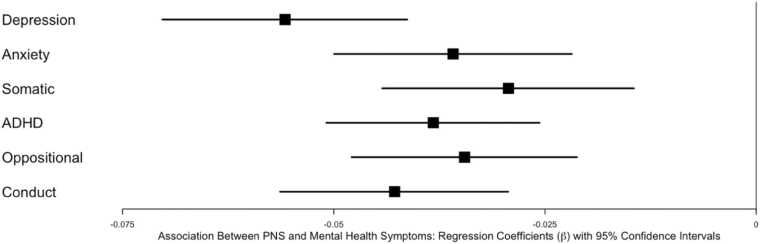
Forest plot illustrating the relationship between perceived neighborhood safety (PNS) and mental health outcomes. Each dot represents the beta coefficient from linear mixed-effects models, with bars indicating the 95 % confidence intervals. Models accounted for familial and neighborhood poverty as well as population density. Objective neighborhood violence was not significantly associated with mental health outcomes and is not displayed in this figure.

**Fig. 2:**
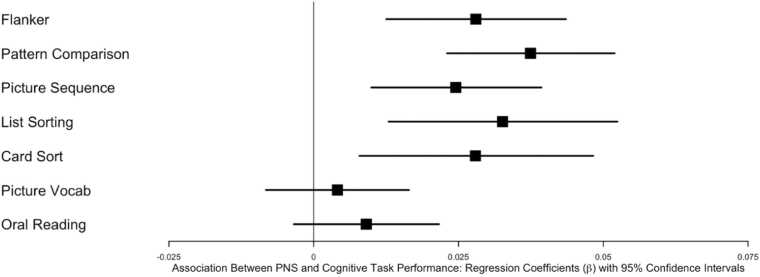
Forest plot illustrating the relationship between perceived neighborhood safety (PNS) and cognitive performance. Each dot represents the beta coefficient from linear mixed-effects models, with bars indicating the 95 % confidence intervals. Models accounted for familial and neighborhood poverty as well as population density. Objective neighborhood violence was not significantly associated with cognitive outcomes and is not displayed in this figure.

To examine the relationship between neighborhood violence and mental health and cognition, we used linear mixed-effects models accounting for familial and neighborhood poverty as well as population density. As shown in Table 2, objective neighborhood violence was not predictive of mental health or cognition after Bonferroni correction. However, as shown in Table 2, PNS predicted mental health outcomes across the board. Youth who reported feeling safer in their neighborhoods had fewer caregiver-reported depressive problems, anxiety problems, somatic problems, ADHD problems, oppositional defiant problems, and conduct disorder problems (see Fig. 1). Regarding cognition, youth who reported feeling safer in their neighborhood performed better on NIH Toolbox Flanker, NIH Toolbox List Sorting, NIH Toolbox Pattern Comparison, and NIH Toolbox Picture Sequence (see Fig. 2).

**Table 2:** Associations Between Objective Neighborhood Violence and Perceived Neighborhood Safety and Mental Health and Cognitive Outcomes.

	Objective Neighborhood Violence				Perceived Neighborhood Safety			
	Mental Health							
Outcome	Estimate(β)	SE	t	p	Estimate(β)	SE	t	p
Depression	-0.0235	0.0134	-1.11	0.275	-0.0558	0.0074	-7.55	< .001*
Anxiety	-0.0307	0.0217	-1.42	0.164	-0.0359	0.0072	-5.00	< .001*
Somatic	-0.0023	0.0203	-0.11	0.909	-0.0293	0.0076	-3.87	< .001*
ADHD	-0.0273	0.0219	-1.25	0.219	-0.0382	0.0064	-5.95	< .001*
Oppositional	-0.0244	0.0176	-1.38	0.176	-0.0345	0.0068	-5.01	< .001*
Conduct Disorder	-0.0367	0.0214	-1.79	0.076	-0.0428	0.0070	-6.60	< .001*
	Cognition							
Outcome	Estimate(β)	SE	t	p	Estimate(β)	SE	t	p
Flanker	0.0470	0.0223	2.11	0.041	0.0280	0.0079	3.54	< .001*
Pattern Comparison	0.0094	0.0241	0.39	0.698	0.0374	0.0074	5.07	< .001*
Picture Sequencing	0.0324	0.0135	2.40	0.023	0.0245	0.0075	3.28	0.001*
List Sort	0.0346	0.0205	1.69	0.100	0.0326	0.0101	3.24	0.001*
Card Sort	-0.0301	0.0197	1.56	0.128	0.0279	0.0103	2.72	0.007*
Picture Vocabulary	0.0535	0.0256	2.09	0.042	0.0058	0.0063	0.92	0.358
Oral Reading	0.0534	0.0219	2.43	0.020	0.0091	0.0064	1.42	0.155

* indicates significance after Bonferroni correction (p < 0.008 for mental health; p < .007 for cognition).

### 4.4 Relationship of perceived neighborhood safety to cognition and mental health outcomes, when controlling for BIS

To examine the possibility that personality/temperament traits related to neuroticism drive perceptions of neighborhood safety, we again used linear mixed-effects models to investigate the relationship between PNS and mental health and cognition, while controlling for BIS scores. All of our previously observed associations remained significant even when controlling for BIS (see Supplemental Table 22).

### 4.5 Concurrent and Longitudinal Relationships Between BIS and PNS

To examine the relationship between BIS and PNS, a linear mixed effects model was used to determine if BIS and PNS are concurrently associated with each other. Increased PNS scores were found to be significantly associated with increased BIS scores (β = −0.0758, p < .001). To examine if baseline BIS was associated with year 2 PNS, when controlling for baseline BIS, a linear mixed effects model was used. Baseline BIS was not found to be associated with change in PNS scores from baseline to year 2 (β = −0.0225, p = 0.0491) after Bonferroni correction (p < 0.025). Additionally, to determine if there was an association between baseline PNS and year 2 BIS scores, when controlling for baseline BIS, a linear mixed effects model was used. Baseline PNS was not found to be significantly associated with the change in BIS scores from baseline to year 2 (β = −0.0074, p = 0.55).

### 4.6 Relationship of perceived neighborhood safety to cognition and mental health outcomes, when controlling for self-reported direct violence exposure

To better understand if direct violence exposure might be playing a role in the observed associations between PNS and mental health and cognition, linear mixed effects models were used with mental health and cognition variables as the dependent variables and PNS as the independent variable, controlling for self-reported direct violence exposure. All of our previously observed associations remained significant even when controlling for self-reported direct violence exposure (see Supplemental Table 23).

### 4.7 Perceived neighborhood safety as a possible moderator of the association between neighborhood violence exposure and adolescent mental health and cognitive outcomes

To investigate whether PNS moderates the relationship between neighborhood violence (NV) and mental health or cognitive outcomes in adolescents, we employed linear mixed-effects models. We tested this hypothesis using 13 separate models, adjusting for covariates such as sex, interview age, site, and familial status. Across all 13 models, no significant moderation by PNS was found in the relationship between objective neighborhood violence and mental health or cognitive outcomes (see Table 3).

**Table 3:** Associations Between the Interaction of Perceived Neighborhood Safety and Objective Neighborhood Violence and Mental Health and Cognitive Outcomes.

Mental Health				
Moderation Outcome	Estimate(β) value for interaction	SE	t value for interaction	p value for interaction
Depression	0.007	0.015	0.45	0.509
Anxiety	-0.008	0.014	-0.54	0.591
Somatic	-0.025	0.015	-1.72	0.086
ADHD	0.019	0.014	1.38	0.167
Oppositional	0.011	0.014	0.82	0.411
Conduct	0.025	0.013	1.89	0.059
Cognition				
Flanker	0.005	0.008	0.58	0.560
Pattern Comparison	-0.014	0.008	-1.83	0.067
Picture Sequencing	0.011	0.008	1.43	0.153
List Sorting	0.001	0.011	0.09	0.930
Card Sort	-0.006	0.010	-0.63	0.531
Picture Vocabulary	-0.002	0.007	-0.33	0.738
Oral Reading	0.002	0.007	0.36	0.719

### 4.8 Perceived safety and related brain areas

To examine the relationship between PNS and brain areas frequently associated with stress responses, we employed seven linear mixed effects models (one for each brain area, superior pre-frontal cortex, medial prefrontal cortex, inferior prefrontal cortex, left hippocampus, right hippocampus, left amygdala, and right amygdala) each controlling for intracranial volume, county-wide neighborhood crime prevalence, population density, area deprivation index, family income, sex and age. No significant relationship was found between PNS and the pre-frontal cortex, hippocampus, or left amygdala (Table 4). Increased PNS was significantly associated with increased right amygdala volume after applying Bonferroni Correction (Table 4).

**Table 4:** Associations Between Perceived Neighborhood Safety and Brain Volumes.

Brain Area	Estimate	SE	t value	p
Right Amygdala	0.013	0.005	2.621	0.006*
Left Amygdala	0.005	0.005	1.192	0.235
Right Hippocampus	0.003	0.003	0.967	0.333
Left Hippocampus	0.002	0.003	0.538	0.591
Superior PFC	0.002	0.002	0.766	0.443
Medial PFC	0.001	0.003	0.121	0.903
Inferior PFC	0.002	0.003	0.660	0.509

* indicates significance after Bonferroni correct (p < .007)

### 4.9 Brain areas and related outcomes

To determine if the right amygdala is involved the same outcomes associated with PNS we utilized 10 linear mixed effects models (6 CBCL DSM scales for depression problems, anxiety problems, somatic problems, ADHD problems, oppositional defiant problems, conduct problems; four NIH Toolbox Cognitive Games including Flanker, Pattern Comparison, Picture Sequence, and List Sorting) each accounting for PNS, county-wide violent crime prevalence, population density, area deprivation index, family income, intracranial volume, sex, and age. No significant relationship was seen between the right amygdala and the mental health outcomes or cognitive outcomes after Bonferroni correction (Supplemental Table 1).

### 4.10 Longitudinal investigation into possible directionality between PNS and mental health and cognition

To investigate the potential directionality between PNS and mental health and cognition, we utilized the longitudinal design of the ABCD study. To determine if baseline PNS predicted mental health or cognitive outcomes at year two, we conducted linear mixed effects models controlling for baseline mental health and cognition. These models also accounted for county-wide violent crime prevalence, population density, area deprivation index, family income, sex, and age. As shown in Table 5, a significant association was observed between greater baseline PNS and increased year two Picture Sequencing performance after controlling for baseline Picture Sequencing performance. No other significant associations were found between baseline PNS and year two mental health outcomes or cognitive outcomes after Bonferroni correction (Table 5) when controlling for baseline mental health or cognition.

**Table 5:** Longitudinal Associations Between Perceived Neighborhood Safety and Mental Health and Cognitive Outcomes.

Perceived Neighborhood Safety At Baseline Predicting Mental Health and Cognition Outcomes at Year two							
Outcome	Estimate		SE		t value	p	
Depression	-0.025		0.011		-2.40	0.016	
Anxiety	-0.007		0.010		-0.71	0.476	
Somatic	-0.017		0.012		-1.409	0.159	
ADHD	-0.019		0.009		-2.14	0.032	
Oppositional	-0.017		0.009		-1.75	0.080	
Conduct	-0.025		0.010		-2.52	0.012	
Flanker	0.004		0.012		0.360	0.719	
Pattern Comparison	0.004		0.011		0.317	0.751	
Picture Sequence	0.027		0.010		2.654	0.007*	
Mental Health and Cognition at Baseline Predicting Perceived Neighborhood Safety at Year two							
Predictor		Estimate		SE	t value		p
Depression	-0.029		0.010		-2.93	0.003*	
Anxiety	-0.037		0.010		-3.80	< .001*	
Somatic	-0.045		0.202		-4.597	< .001*	
ADHD	-0.063		0.010		-6.26	< .001*	
Oppositional	-0.047		0.010		-4.74	< .001*	
Conduct	-0.054		0.011		-5.10	< .001*	
Flanker	0.009		0.010		0.92	0.358	
Pattern Comparison	0.010		0.010		0.99	0.320	
Picture Sequencing	0.013		0.010		1.30	0.192	
List Sorting	0.032		0.010		3.15	0.002*	
Card Sort	0.013		0.010		1.33	0.182	

* indicates significance after Bonferroni correction (p < .008 for mental health as an outcome; p < .0.017 for cognition as an outcome; p < .008 for mental health as a predictor; p < 0.01 for cognition as a predictor)

To determine if baseline mental health or cognition predicted PNS at year two, we conducted linear mixed effects models controlling for baseline PNS. These models also accounted for county-wide violent crime prevalence, population density, area deprivation index, family income, sex, and age. As shown in Table 5, after Bonferroni correction, a significant relationship was found between higher scores on all 6 CBCL DSM mental health subscales at baseline and lower PNS at year two, when controlling for baseline PNS and all of the other model predictors. A significant association was found between greater baseline NIH Toolbox List Sorting performance and increased year two PNS, controlling for baseline PNS. No other significant relations were identified between baseline cognitive task performance and year two PNS, when controlling for baseline PNS.

## 5 Discussion

The goal of this study was to examine the relationship between children’s perceptions of neighborhood safety and outcomes related to mental health, cognition, and brain structure. While previous work has often focused on objective indicators of neighborhood violence, our findings highlight the importance of subjective experience in shaping developmental outcomes. Perceived neighborhood safety appeared to carry an independent association with both mental health symptomology and cognitive functioning. This distinction raises questions about the mechanisms by which environmental factors influence development–particularly when perceptions diverge from objective conditions–and emphasizes the need to better understand the factors associated with youth’s interpretations and internalizations of their surroundings.

This study examined how neighborhood violence and perceptions of neighborhood safety are related to psychopathology risk and cognitive functioning. To do so, we first examined the factors that predict perceived neighborhood safety beyond objective neighborhood violence. Our findings indicate that children from more resourced environments at a familial and neighborhood level, who report greater community cohesion, who have lower caregiver reported psychopathology, and who are from less populated areas perceive their neighborhoods as being safer. Conversely, higher perceived neighborhood safety is associated with to greater combined family income and stronger community cohesion. Notably, objective neighborhood violence was not significantly associated with perceived neighborhood safety in our model. These results suggest that socioeconomic and community factors shape perceptions of safety more than objective crime rates.

We initially hypothesized that objective neighborhood violence would predict increased mental health symptoms and poorer cognitive functioning, and that perceived neighborhood safety would moderate this relationship–specifically, that higher perceived neighborhood safety would weaken the negative effects of neighborhood violence on mental health and cognition. However, this hypothesis was not supported. Objective neighborhood violence was not significantly associated with mental health or cognitive outcomes either as a main effect or as moderated by perceived neighborhood safety. Instead, perceived neighborhood safety itself was significantly associated with both increased mental health symptoms and decreased cognitive performance. These results align with findings from the meta- analysis conducted by Baranyi et al. (2021) that subjective experiences of neighborhood threat often exert a stronger influence on mental health and cognitive outcomes than objective environmental factors.

The present findings suggest that perceived neighborhood safety is more strongly associated with fluid cognitive abilities than with crystallized cognition, a pattern that may reflect the differential sensitivity of these domains to environmental stressors. Fluid cognitive skills, which encompasses executive function, working memory, and attentional control, is particularly vulnerable to chronic stress and threat-related vigilance, as these processes rely on efficient prefrontal cortex functioning (Stawski et al., 2010, Woo et al., 2021). In contrast, crystallized abilities, such as verbal knowledge and general information retention, are more dependent on accumulated learning over time and tend to be less susceptible to acute or chronic environmental stressors and threat (Friedel et al., 2015, Sand et al., 2024, Shields et al., 2017, Woo et al., 2021). Prior work has demonstrated that individuals who perceive their neighborhoods as unsafe show deficits in working memory and executive functioning, but not in tasks requiring well-rehearsed verbal knowledge (Muñoz et al., 2020), and our findings are consistent with this pattern.

These differential associations with fluid versus crystallized cognition may stem from the cognitive load imposed by persistent safety concerns, which can deplete attentional resources necessary for fluid cognitive processes while leaving crystallized knowledge relatively intact. Moreover, there is evidence that neighborhood adversity relations to cognition appear to be moderated by social and demographic factors, including sex and quality of school environment, suggesting that broader structural inequalities likely shape these associations (Rakesh et al., 2021). Given the established links between cognitive function and broader mental health outcomes, future research should examine whether the relationship of neighborhood perceptions to fluid cognition contributes to later emotional or psychiatric risk.

Given prior research implicating the HPA axis in threat perception and its downstream effects on mental health and cognition (Ferrer et al., 2020, Gardner et al., 2019, Mikulska et al., 2021), we examined whether structural differences in key brain regions were associated with perceived neighborhood safety. Specifically, we investigated the left amygdala, right amygdala, left hippocampus, right hippocampus, superior prefrontal cortex, medial prefrontal cortex, and the inferior prefrontal cortex. We found that greater perceived neighborhood safety was significantly associated with right amygdala volume, while no significant associations were observed for the other brain regions. This finding aligns with prior research linking stress exposure to reduced right amygdala volume (C. H. Fowler et al., 2021). The observed difference in right amygdala volume associated with PNS may also complement the finding of increased right amygdala threat reactivity in those reporting lower perceptions of neighborhood safety (Suarez et al., 2022). These findings fit into the broader set of adversity-related literature suggesting amygdala volumetric changes associated with stress as a function of excitotoxicity (Hanson and Nacewicz, 2021). However, the right amygdala was not significantly associated with cognitive or mental health outcomes, suggesting that while this region may play a role in perceived safety, we do not find evidence that it mediates the relationship between perceived safety and broader mental health symptomology or cognitive effects.

Our original hypotheses were derived from the idea that perceived neighborhood safety contributed to mental health concerns above measures of objective crime. However, our findings indicate that baseline mental health significantly predicted year two perceived neighborhood safety, even after accounting for baseline perceptions of safety. This suggests that mental health symptoms may shape how individuals experience their environments over time, rather than perceptions of safety solely influencing mental health outcomes. Individuals with higher levels of anxiety or depression may become more attuned to potential threats in their surroundings, consistent with prior research showing that internalizing symptoms are associated with heightened threat sensitivity and negative cognitive biases (Beevers et al., 2015, Grupe and Nitschke, 2013). This pattern aligns with cognitive models of psychopathology, which propose that emotional distress can alter attentional and interpretative processes, leading individuals to situations as more dangerous regardless of objective threat (Beevers et al., 2015, Grupe and Nitschke, 2013). While our findings differ from prior research linking perceived neighborhood safety to later mental health and cognitive concerns, this discrepancy may be explained by the inclusion of covariates such as the Area Deprivation Index and familial combined income. These factors, which have been shown in previous studies to influence mental health (Kinge et al., 2021, Visser et al., 2021), were also significant predictors of year two PNS when controlling for baseline PNS (See supplemental tables 2–21). Thus, previous studies that did not include measures such as area deprivation or family income (Kinge et al., 2021, Visser et al., 2021) may have seen relationships between perceived neighborhood safety and mental health that reflected their shared associations with these “third” variables. This pattern suggests that socioeconomic factors rather than perceived safety itself, may be driving the increase in mental health symptoms.

We did not observe the same pattern of findings for cognition, as most measures of baseline cognitive function were not predictive of changes in perceived neighborhood safety over time. Increased baseline PNS was significantly associated with better year two picture sequencing performance, a measure of episodic memory, when controlling for baseline picture sequence performance. This finding points to episodic memory function as potentially being vulnerable to diminished PNS, which aligns with previous research indicating that stress may disrupt episodic memory processes (Sherman et al., 2024, Shields et al., 2017). PNS did not significantly predict the change in function from baseline to year two for any of the other cognitive domains. One possible explanation is that cognitive abilities, particularly fluid cognition, are less directly tied to threat detection and emotional processing than mental health symptoms (Stawski et al., 2010). Additionally, baseline performance on the list sorting task was significantly associated with year two PNS after controlling for baseline PNS. This suggests that in addition to mental health concerns, working memory may also play a role in shaping neighborhood safety perception, pointing to the previously established role of working memory in attentional biases and threat perception (Crick and Dodge, 1994, Satmarean et al., 2022, Wyche et al., 2024). Besides the relationship observed with baseline list sorting performance, no significant association was found between any other baseline cognition measure and year two PNS, when controlling for baseline PNS. However, it should be noted that we cannot rule out the possibility that there is a more predictive relationship from perceived neighborhood safety to later cognitive function emerges earlier in development. During early childhood, the brain regions involved in attention, working memory, and emotion regulation undergo rapid development and may be more sensitive to environmental inputs such as perceived threat or safety (Burris et al., 2022, Luby et al., 2021, Perlman and Pelphrey, 2010).This may make the early years a more critical window for PNS to influence cognitive outcomes. While an association between cognition and PNS was observed, further and possibly earlier longitudinal investigation would be necessary to clarify whether PNS influences cognitive development, whether cognitive abilities shape perceptions of safety, or whether both are shaped by shared early-life contextual factors.

To determine whether observed associations between perceived neighborhood safety (PNS) and youth outcomes were accounted for by personality/temperament traits related to neuroticism or by direct violence exposure, we re-ran all models including the Behavioral Inhibition System (BIS) scale and a composite of direct violence exposure as covariates. In both cases, all previously observed associations between PNS and mental health and cognition remained significant (see Supplemental Tables 22–23). Although PNS and BIS were concurrently related, BIS did not predict change in PNS over time, suggesting that PNS captures unique variance in mental health and cognitive functioning beyond neuroticism and direct exposure to violence.

This study includes several limitations. First, because we examined these relationships in children aged 9–12, it is possible we are not capturing the developmental window most sensitive to the effects of perceived neighborhood safety (PNS); a similar study in younger children may reveal a different set of associations. Second, our measure of PNS consisted of a single Likert-rated item. While this approach has precedent, a multi-item or composite measure may better capture the complexity of this construct. Third, we attempted to adjust for population density in measuring objective neighborhood violence by including it as a covariate. However, population density was reported at the census tract level, while violence data were reported at the county level. This mismatch in granularity may have introduced discrepancies in how neighborhood-level conditions were represented for each participant.

In summary, our results suggest that factors beyond objective neighborhood violence– including family income and area-level deprivation–are linked to how safe children feel in their neighborhoods. In turn, these perceptions appear to relate to both mental health and cognitive outcomes, whereas objective violence itself does not. Interestingly, our findings suggest that mental health symptoms may precede lower perceptions of safety, raising the possibility that internal states may shape how young people interpret their environments. We also found that reduced PNS is associated with smaller right amygdala volume, a brain region central to threat detection, suggesting that children’s sense of safety may be tied to structural differences in fear-related neural systems. Together, these findings highlight the importance of understanding children’s perceptions of safety–both in terms of what shapes them and how they, in turn, may impact development.

## CRediT authorship contribution statement

**Nourhan M. Elsayed:** Writing – review & editing, Supervision, Methodology, Investigation, Formal analysis, Conceptualization. **Deanna M. Barch:** Writing – review & editing, Supervision, Methodology, Investigation. **Lindsley Patrick M:** Writing – review & editing, Writing – original draft, Visualization, Methodology, Investigation, Formal analysis, Conceptualization.

## Declaration of Competing Interest

The authors declare no known financial or personal conflicts of interest that could have influenced the work detailed in this paper.

## Data Availability

Data will be made available on request.
