# Supplementary material for: Contributions of neighborhood violent crime and perceived neighborhood safety to cognition and mental health in the adolescent brain cognitive development study
Source: Dev Cogn Neurosci. 2025 Dec 18;77:101660. doi: 10.1016/j.dcn.2025.101660 (PMC12775686; doi:10.1016/j.dcn.2025.101660)
Supplement: Supplementary file 1 — Supplementary material [file mmc1.docx]

**Supplemental Materials**

**Pre-Registration Deviations**

This project was pre-registered to examine (1) factors beyond neighborhood violence that may contribute to perceived neighborhood safety, (2) associations between neighborhood violence and both mental health and cognitive outcomes, and (3) potential interactions between objective neighborhood violence and perceived neighborhood safety in relation to mental health and cognition. While the pre-registration outlined a longitudinal approach, specifically, using baseline perceived neighborhood safety (PNS) to predict year two outcomes, we ultimately chose to conduct all of the originally planned analyses concurrently, still including baseline and year two timepoints. This decision was made because we were not testing a specific developmental trajectory that would necessitate longitudinal modeling.

We also deviated from the original plan for operationalizing objective neighborhood violence. Initially, we intended to calculate the rate of violent crime per census tract reported population to account for population density. However, given prior evidence suggesting that population density itself may be associated with mental health and cognitive outcomes, we opted to include both neighborhood violent crime and census tract population as separate predictors. This approach allowed us to better isolate the unique contribution of each factor. Additionally, census tract population was windosrized, due to extreme outliers seemingly driven by factors that inflate population density measures (e.g., public parks, airports).

All other planned analyses were conducted as pre-registered.

In addition to these planned analyses, we pursued follow-up tests to better understand an unexpected pattern: the relationship between PNS and mental health/cognition. We explored associations between perceived safety and brain regions involved in threat response (notably those linked to HPA axis functioning), as well as whether baseline PNS predicted year two mental health and cognition when controlling for baseline outcomes. We also ran the inverse model, examining whether baseline mental health and cognition predicted year two PNS, to better understand the directionality of these associations.

**Supplemental Table 1: Right Amygdala Predicting Mental Health and Cognition Outcomes**

| Mental Health | | | | |
| --- | --- | --- | --- | --- |
| Moderation Outcome | Estimate(β) | SE | t value | p |
| Depression | -0.007 | 0.010 | -0.67 | 0.505 |
| Anxiety | 0.002 | 0.011 | 0.159 | 0.874 |
| Somatic | -0.013 | 0.011 | -1.22 | 0.221 |
| ADHD | -0.007 | 0.011 | -0.66 | 0.510 |
| Oppositional | -0.023 | 0.011 | -2.04 | 0.042 |
| Conduct | -0.023 | 0.012 | -1.96 | 0.050 |
| Cognition | | | | |
| Flanker | 0.004 | 0.011 | 0.35 | 0.724 |
| Pattern Comparison | -0.005 | 0.012 | -0.40 | 0.692 |
| Picture Sequence | 0.001 | 0.011 | 0.62 | 0.950 |
| List Sorting | 0.029 | 0.012 | 2.36 | 0.020 |
| Card Sort | 0.004 | 0.147 | 0.02 | 0.980 |

**Supplemental Table 2: Association Between Baseline Perceived Neighborhood Safety (PNS) and Year 2 Depression Problems Accounting for Baseline Depression Problems**

| Variable | Estimate(β) | SE | t value | p |
| --- | --- | --- | --- | --- |
| Baseline PNS | -0.025 | 0.011 | -2.400 | 0.016 |
| Objective Neighborhood Violence | -0.023 | 0.017 | -1.353 | 0.183 |
| Population Density | 0.010 | 0.013 | 0.738 | 0.460 |
| Baseline Depression Problems | 0.543 | 0.010 | 54.480 | < 2e-16 * |
| Sex (effect coded) | 0.057 | 0.020 | 2.894 | 0.004 * |
| Age | 0.036 | 0.010 | 3.640 | < .001 * |
| Family Income | -0.023 | 0.013 | -1.772 | 0.076 |
| Area Deprivation Index (Reverse Coded) | 0.032 | 0.014 | 2.263 | 0.024 |

* Indicates significance after Bonferroni correction (p < .008)

**Supplemental Table 3: Association Between Baseline Perceived Neighborhood Safety (PNS) and Year 2 Anxiety Problems Accounting for Baseline Anxiety Problems**

| Variable | Estimate(β) | SE | t value | p |
| --- | --- | --- | --- | --- |
| Baseline PNS | -0.007 | 0.010 | -0.713 | 0.476 |
| Objective Neighborhood Violence | 0.000 | 0.014 | -0.006 | 0.995 |
| Population Density | -0.001 | 0.013 | -0.065 | 0.949 |
| Baseline Anxious Problems | 0.608 | 0.010 | 62.218 | < 2e-16 * |
| Sex (effect coded) | 0.074 | 0.019 | 3.888 | < .001 * |
| Age | -0.006 | 0.010 | -0.677 | 0.498 |
| Family Income | -0.016 | 0.012 | -1.317 | 0.188 |
| Area Deprivation Index (Reverse Coded) | 0.035 | 0.014 | 2.550 | 0.011 |

* Indicates significance after Bonferroni correction (p < .008)

**Supplemental Table 4: Association Between Baseline Perceived Neighborhood Safety (PNS) and Year 2 Somatic Problems Accounting for Baseline Somatic Problems**

| Variable | Estimate(β) | SE | t value | p |
| --- | --- | --- | --- | --- |
| Baseline PNS | -0.017 | 0.012 | -1.409 | 0.159 |
| Objective Neighborhood Violence | -0.011 | 0.016 | -0.654 | 0.516 |
| Population Density | -0.028 | 0.015 | -1.882 | 0.060 |
| Baseline Somatic Problems | 0.482 | 0.012 | 41.844 | <2e-16 * |
| Sex (effect coded) | 0.049 | 0.023 | 2.160 | 0.031 |
| Age | -0.014 | 0.011 | -1.223 | 0.222 |
| Family Income | -0.010 | 0.015 | -0.685 | 0.493 |
| Area Deprivation Index (Reverse Coded) | -0.013 | 0.016 | -0.822 | 0.411 |

* Indicates significance after Bonferroni correction (p < .008)

**Supplemental Table 5: Association Between Baseline Perceived Neighborhood Safety (PNS) and Year 2 ADHD Problems Accounting for Baseline ADHD Problems**

| Variable | Estimate(β) | SE | t value | p |
| --- | --- | --- | --- | --- |
| Baseline PNS | -0.019 | 0.009 | -2.141 | 0.032 |
| Objective Neighborhood Violence | -0.014 | 0.014 | -0.988 | 0.329 |
| Population Density | -0.011 | 0.011 | -0.987 | 0.324 |
| Baseline ADHD Problems | 0.710 | 0.009 | 82.209 | < 2e-16 * |
| Sex (effect coded) | -0.089 | 0.017 | -5.283 | < .001 * |
| Age | -0.001 | 0.008 | -0.127 | 0.899 |
| Family Income | 0.001 | 0.011 | 0.059 | 0.953 |
| Area Deprivation Index (Reverse Coded) | 0.011 | 0.012 | 0.898 | 0.369 |

* Indicates significance after Bonferroni correction (p < .008

**Supplemental Table 6: Association Between Baseline Perceived Neighborhood Safety (PNS) and Year 2 Oppositional Defiant Problems Accounting for Baseline Oppositional Defiant Problems**

| Variable | Estimate(β) | SE | t value | p |
| --- | --- | --- | --- | --- |
| Baseline PNS | -0.017 | 0.010 | -1.751 | 0.080 |
| Objective Neighborhood Violence | -0.013 | 0.012 | -1.133 | 0.261 |
| Population Density | -0.006 | 0.012 | -0.479 | 0.632 |
| Baseline Oppositional Defiant Problems | 0.653 | 0.009 | 71.498 | <2e-16 * |
| Sex (effect coded) | -0.045 | 0.018 | -2.471 | 0.014 |
| Age | 0.020 | 0.009 | 2.198 | 0.028 |
| Family Income | -0.011 | 0.012 | -0.953 | 0.341 |
| Area Deprivation Index (Reverse Coded) | -0.010 | 0.013 | -0.794 | 0.428 |

* Indicates significance after Bonferroni correction (p < .008)

**Supplemental Table 7: Association Between Baseline Perceived Neighborhood Safety (PNS) and Year 2 Conduct Problems Accounting for Baseline Conduct Problems**

| Variable | Estimate(β) | SE | t value | p |
| --- | --- | --- | --- | --- |
| Baseline PNS | -0.025 | 0.010 | -2.515 | 0.012 |
| Objective Neighborhood Violence | -0.013 | 0.013 | -1.013 | 0.012 |
| Population Density | -0.015 | 0.012 | -1.264 | 0.207 |
| Baseline Conduct Problems | 0.617 | 0.010 | 61.733 | < 2e-16 * |
| Sex (effect coded) | -0.047 | 0.018 | -2.525 | 0.012 |
| Age | 0.026 | 0.009 | 2.829 | 0.005 * |
| Family Income | -0.054 | 0.012 | -4.511 | < .001 * |
| Area Deprivation Index (Reverse Coded) | 0.004 | 0.013 | 0.286 | 0.775 |

* Indicates significance after Bonferroni correction (p < .008)

**Supplemental Table 8: Association Between Baseline Perceived Neighborhood Safety (PNS) and Year 2 Flanker Inhibitory Control Performance Accounting for Baseline Flanker Inhibitory Control Performance**

| Variable | Estimate(β) | SE | t value | p |
| --- | --- | --- | --- | --- |
| Baseline PNS | 0.004 | 0.012 | 0.360 | 0.719 |
| Objective Neighborhood Violence | 0.018 | 0.022 | 0.791 | 0.719 |
| Population Density | 0.003 | 0.015 | 0.173 | 0.863 |
| Baseline Flanker | 0.366 | 0.011 | 33.253 | < 2e-16 * |
| Sex (effect coded) | -0.045 | 0.022 | -2.069 | 0.039 |
| Age | -0.092 | 0.011 | -8.449 | < 2e-16 * |
| Family Income | 0.087 | 0.014 | 6.183 | < .001 * |
| Area Deprivation Index (Reverse Coded) | 0.058 | 0.016 | 3.691 | < .001 * |

* Indicates significance after Bonferroni correction (p < .01)

**Supplemental Table 9: Association Between Baseline Perceived Neighborhood Safety (PNS) and Year 2 Pattern Comparison Performance Accounting for Baseline Pattern Comparison Performance**

| Variable | Estimate(β) | SE | t value | p |
| --- | --- | --- | --- | --- |
| Baseline PNS | 0.004 | 0.011 | 0.317 | 0.751 |
| Objective Neighborhood Violence | 0.044 | 0.019 | 2.289 | 0.028 |
| Population Density | 0.041 | 0.015 | 2.823 | 0.005 * |
| Baseline Pattern Comparison | 0.438 | 0.011 | 40.099 | < 2e-16 * |
| Sex (effect coded) | 0.131 | 0.021 | 6.235 | < .001 * |
| Age | 0.038 | 0.011 | 3.515 | < .001 * |
| Family Income | 0.080 | 0.014 | 5.869 | < .001 * |
| Area Deprivation Index (Reverse Coded) | 0.062 | 0.015 | 4.107 | < .001 * |

* Indicates significance after Bonferroni correction (p < .01)

**Supplemental Table 10: Association Between Baseline Perceived Neighborhood Safety (PNS) and Year 2 Picture Sequence Performance Accounting for Baseline Picture Sequence Performance**

| Variable | Estimate(β) | SE | t value | p |
| --- | --- | --- | --- | --- |
| Baseline PNS | 0.027 | 0.010 | 2.654 | 0.007 * |
| Objective Neighborhood Violence | 0.008 | 0.015 | 0.516 | 0.609 |
| Population Density | 0.007 | 0.013 | 0.568 | 0.570 |
| Baseline Picture Sequence | 0.378 | 0.010 | 38.922 | < 2e-16 * |
| Sex (effect coded) | 0.060 | 0.019 | 3.124 | 0.002 * |
| Age | 0.110 | 0.010 | 11.543 | < 2e-16 * |
| Family Income | 0.101 | 0.012 | 8.225 | < .001 * |
| Area Deprivation Index (Reverse Coded) | 0.057 | 0.014 | 4.150 | < .001 * |

* Indicates significance after Bonferroni correction (p < .01)

**Supplemental Table 11: Association Between Baseline Depression Problems and Year 2 Perceived Neighborhood Safety (PNS) Accounting for Baseline Perceived Neighborhood Safety (PNS)**

| Variable | Estimate(β) | SE | t value | p |
| --- | --- | --- | --- | --- |
| Baseline Depression Problems | -0.029 | 0.010 | -2.932 | 0.664 |
| Objective Neighborhood Violence | -0.016 | 0.020 | -0.776 | 0.003 * |
| Population Density | -0.069 | 0.014 | -5.055 | < .001 * |
| Baseline PNS | 0.207 | 0.010 | 19.812 | < 2e-16 * |
| Sex (effect coded) | -0.031 | 0.019 | -1.622 | 0.105 |
| Age | -0.024 | 0.010 | -2.502 | 0.012 |
| Family Income | 0.063 | 0.012 | 5.039 | < .001 * |
| Area Deprivation Index (Reverse Coded) | 0.202 | 0.014 | 14.381 | < 2e-16 * |

* Indicates significance after Bonferroni correction (p < .008)

**Supplemental Table 12: Association Between Baseline Anxiety Problems and Year 2 Perceived Neighborhood Safety (PNS) Accounting for Baseline Perceived Neighborhood Safety (PNS)**

| Variable | Estimate(β) | SE | t value | p |
| --- | --- | --- | --- | --- |
| Baseline Anxiety Problems | -0.037 | 0.010 | -3.798 | < .001 * |
| Objective Neighborhood Violence | -0.017 | 0.020 | -0.840 | 0.407 |
| Population Density | -0.069 | 0.014 | -5.035 | < .001 * |
| Baseline PNS | 0.206 | 0.010 | 19.809 | < 2e-16 * |
| Sex (effect coded) | -0.028 | 0.019 | -1.424 | 0.154 |
| Age | -0.025 | 0.010 | -2.577 | 0.010 |
| Family Income | 0.063 | 0.012 | 5.095 | < .001 * |
| Area Deprivation Index (Reverse Coded) | 0.203 | 0.014 | 14.422 | < 2e-16 * |

* Indicates significance after Bonferroni correction (p < .008)

**Supplemental Table 13: Association Between Baseline Somatic Problems and Year 2 Perceived Neighborhood Safety (PNS) Accounting for Baseline Perceived Neighborhood Safety (PNS)**

| Variable | Estimate(β) | SE | t value | p |
| --- | --- | --- | --- | --- |
| Baseline Somatic Problems | -0.045 | 0.202 | -4.597 | < .001 * |
| Objective Neighborhood Violence | -0.015 | 0.020 | -0.770 | 0.447 |
| Population Density | -0.069 | 0.014 | -5.022 | < .001 * |
| Baseline PNS | 0.206 | 0.010 | 19.839 | < 2e-16 * |
| Sex (effect coded) | -0.024 | 0.019 | -1.224 | 0.221 |
| Age | -0.023 | 0.010 | -2.423 | 0.015 |
| Family Income | 0.063 | 0.012 | 5.090 | < .001 * |
| Area Deprivation Index (Reverse Coded) | 0.202 | 0.014 | 14.406 | < 2e-16 * |

* Indicates significance after Bonferroni correction (p < .008)

**Supplemental Table 14: Association Between Baseline ADHD Problems and Year 2 Perceived Neighborhood Safety (PNS) Accounting for Baseline Perceived Neighborhood Safety (PNS)**

| Variable | Estimate(β) | SE | t value | p |
| --- | --- | --- | --- | --- |
| Baseline ADHD Problems | -0.063 | 0.010 | -6.258 | < .001 * |
| Objective Neighborhood Violence | -0.016 | 0.020 | -0.812 | 0.423 |
| Population Density | -0.069 | 0.014 | -5.125 | < .001 * |
| Baseline PNS | 0.205 | 0.010 | 19.677 | < 2e-16 * |
| Sex (effect coded) | -0.047 | 0.020 | -2.425 | 0.015 |
| Age | -0.026 | 0.010 | -2.661 | 0.008 |
| Family Income | 0.059 | 0.012 | 4.773 | < .001 * |
| Area Deprivation Index (Reverse Coded) | 0.202 | 0.014 | 14.373 | < 2e-16 * |

* Indicates significance after Bonferroni correction (p < .008)

**Supplemental Table 15: Association Between Baseline Oppositional Defiant Problems and Year 2 Perceived Neighborhood Safety (PNS) Accounting for Baseline Perceived Neighborhood Safety (PNS)**

| Variable | Estimate(β) | SE | t value | p |
| --- | --- | --- | --- | --- |
| Baseline Oppositional Defiant Problems | -0.047 | 0.010 | -4.742 | < .001 * |
| Objective Neighborhood Violence | -0.016 | 0.020 | -0.806 | 0.426 |
| Population Density | -0.069 | 0.014 | -5.071 | < .001 * |
| Baseline PNS | 0.206 | 0.010 | 19.774 | < 2e-16 * |
| Sex (effect coded) | -0.037 | 0.019 | -1.914 | 0.056 |
| Age | -0.025 | 0.010 | -2.645 | 0.008 |
| Family Income | 0.062 | 0.012 | 4.956 | < .001 * |
| Area Deprivation Index (Reverse Coded) | 0.202 | 0.014 | 14.397 | < 2e-16 * |

* Indicates significance after Bonferroni correction (p < .008)

**Supplemental Table 16: Association Between Baseline Conduct Problems and Year 2 Perceived Neighborhood Safety (PNS) Accounting for Baseline Perceived Neighborhood Safety (PNS)**

| Variable | Estimate(β) | SE | t value | p |
| --- | --- | --- | --- | --- |
| Baseline Conduct Problems | -0.054 | 0.011 | -5.102 | < .001 * |
| Objective Neighborhood Violence | -0.015 | 0.020 | -0.786 | 0.437 |
| Population Density | -0.070 | 0.014 | -5.155 | < .001 * |
| Baseline PNS | 0.205 | 0.010 | 19.689 | < 2e-16 * |
| Sex (effect coded) | -0.040 | 0.019 | -2.066 | 0.039 |
| Age | -0.025 | 0.010 | -2.643 | 0.008 |
| Family Income | 0.059 | 0.012 | 4.743 | < .001 * |
| Area Deprivation Index (Reverse Coded) | 0.200 | 0.014 | 14.250 | < 2e-16 * |

* Indicates significance after Bonferroni correction (p < .008)

**Supplemental Table 17: Association Between Baseline Flanker Inhibitory Control Performance and Year 2 Perceived Neighborhood Safety (PNS) Accounting for Baseline Perceived Neighborhood Safety (PNS)**

| Variable | Estimate(β) | SE | t value | p |
| --- | --- | --- | --- | --- |
| Baseline Flanker | 0.009 | 0.010 | 0.920 | 0.358 |
| Objective Neighborhood Violence | -0.018 | 0.020 | -0.905 | 0.372 |
| Population Density | -0.068 | 0.014 | -4.918 | < .001 * |
| Baseline PNS | 0.208 | 0.010 | 19.820 | < 2e-16 * |
| Sex (effect coded) | -0.032 | 0.019 | -1.620 | 0.105 |
| Age | -0.025 | 0.010 | -2.582 | 0.010 |
| Family Income | 0.064 | 0.013 | 5.070 | < .001 * |
| Area Deprivation Index (Reverse Coded) | 0.201 | 0.014 | 14.198 | < 2e-16 * |

* Indicates significance after Bonferroni correction (p < .01)

**Supplemental Table 18: Association Between Baseline Pattern Comparison Performance and Year 2 Perceived Neighborhood Safety (PNS) Accounting for Baseline Perceived Neighborhood Safety (PNS)**

| Variable | Estimate(β) | SE | t value | p |
| --- | --- | --- | --- | --- |
| Baseline Picture Sequence | 0.010 | 0.010 | 0.995 | 0.320 |
| Objective Neighborhood Violence | -0.017 | 0.020 | -0.874 | 0.388 |
| Population Density | -0.068 | 0.014 | -4.913 | < .001 * |
| Baseline PNS | 0.207 | 0.010 | 19.741 | < 2e-16 * |
| Sex (effect coded) | -0.033 | 0.020 | -1.699 | 0.089 |
| Age | -0.025 | 0.010 | -2.581 | 0.010 |
| Family Income | 0.065 | 0.012 | 5.173 | < .001 * |
| Area Deprivation Index (Reverse Coded) | 0.200 | 0.014 | 14.143 | < 2e-16 * |

* Indicates significance after Bonferroni correction (p < .01)

**Supplemental Table 19: Association Between Baseline Picture Sequence Performance and Year 2 Perceived Neighborhood Safety (PNS) Accounting for Baseline Perceived Neighborhood Safety (PNS)**

| Variable | Estimate(β) | SE | t value | p |
| --- | --- | --- | --- | --- |
| Baseline Picture Sequence | 0.013 | 0.010 | 1.304 | 0.192 |
| Objective Neighborhood Violence | -0.018 | 0.020 | -0.879 | 0.386 |
| Population Density | -0.068 | 0.014 | -4.923 | < .001 |
| Baseline PNS | 0.208 | 0.010 | 19.869 | < 2e-16 * |
| Sex (effect coded) | -0.033 | 0.020 | -1.703 | 0.089 |
| Age | -0.025 | 0.010 | -2.593 | 0.010 |
| Family Income | 0.063 | 0.013 | 4.988 | < .001 * |
| Area Deprivation Index (Reverse Coded) | 0.201 | 0.014 | 14.192 | < 2e-16 * |

* Indicates significance after Bonferroni correction (p < .01)

**Supplemental Table 20: Association Between Baseline List Sorting Performance and Year 2 Perceived Neighborhood Safety (PNS) Accounting for Baseline Perceived Neighborhood Safety (PNS)**

| Variable | Estimate(β) | SE | t value | p |
| --- | --- | --- | --- | --- |
| Baseline List Sorting | 0.032 | 0.010 | 3.153 | 0.002 * |
| Objective Neighborhood Violence | -0.017 | 0.020 | -0.868 | 0.391 |
| Population Density | -0.069 | 0.014 | -5.047 | < .001 * |
| Baseline PNS | 0.207 | 0.010 | 19.752 | < 2e-16 * |
| Sex (effect coded) | -0.029 | 0.019 | -1.506 | 0.132 |
| Age | -0.025 | 0.010 | -2.580 | 0.010 |
| Family Income | 0.057 | 0.013 | 4.469 | < .001 * |
| Area Deprivation Index (Reverse Coded) | 0.200 | 0.014 | 14.078 | < 2e-16 * |

* Indicates significance after Bonferroni correction (p < .01)

**Supplemental Table 21: Association Between Baseline Card Sort Performance and Year 2 Perceived Neighborhood Safety (PNS) Accounting for Baseline Perceived Neighborhood Safety (PNS)**

| Variable | Estimate(β) | SE | t value | p |
| --- | --- | --- | --- | --- |
| Baseline Card Sort | 0.013 | 0.010 | 1.334 | 0.182 |
| Objective Neighborhood Violence | -0.017 | 0.020 | -0.875 | 0.388 |
| Population Density | -0.068 | 0.014 | -4.913 | < .001 * |
| Baseline PNS | 0.207 | 0.010 | 19.795 | < 2e-16 * |
| Sex (effect coded) | -0.033 | 0.019 | -1.709 | 0.088 |
| Age | -0.025 | 0.010 | -2.620 | 0.009 |
| Family Income | 0.063 | 0.013 | 5.038 | < .001 * |
| Area Deprivation Index (Reverse Coded) | 0.200 | 0.014 | 14.143 | < 2e-16 * |

* Indicates significance after Bonferroni correction (p < .01)

**Supplementary Table 22: Relationship of Perceived Neighborhood Safety to Cognition and Mental Health Outcomes, When Controlling for BIS**

|  |  | **Outcome** | **beta estimate** | **t value** | **p value** |
| --- | --- | --- | --- | --- | --- |
|  |  | **Perceived Neighborhood Safety (controlling for Behavioral Inhibition)** | | | |
| Mental Health  Bonferroni Corrected p value threshold: p <.008 | | Depression | -0.0452 | -6.35 | <.001* |
|  |  | Anxiety | -0.0324 | -4.70 | <.001* |
|  |  | Somatic | -0.0334 | -4.65 | <.001* |
|  |  | ADHD | -0.0367 | -5.80 | <.001* |
|  |  | Oppositional Defiant | -0.0314 | -4.71 | <.001* |
|  |  | Conduct | -0.0376 | -5.50 | <.001* |
| Cognition  Bonferroni Corrected p value threshold: p <.007 | | Flanker | 0.0315 | 4.15 | <.001* |
|  |  | Pattern Comparison | 0.0392 | 5.53 | <.001* |
|  |  | Picture Sequence | 0.0305 | 4.16 | <.001* |
|  |  | List Sorting | 0.0341 | 3.63 | <.001* |
|  |  | Card Sort | 0.0296 | 3.10 | 0.002* |
|  |  | Picture Vocab | 0.0116 | 1.83 | 0.067 |
|  |  | Oral Reading | 0.0143 | 2.26 | 0.024 |
|  |  | **Behavioral Inhibition (controlling for Perceived Neighborhood Safety)** | | | |
| Mental Health  Bonferroni Corrected p value threshold: p <.008 | | Depression | 0.0686 | 9.52 | <.001* |
|  |  | Anxiety | 0.1063 | 15.17 | <.001* |
|  |  | Somatic | 0.0373 | 5.13 | <.001* |
|  |  | ADHD | 0.0158 | 2.44 | 0.015 |
|  |  | Oppositional Defiant | 0.0048 | 0.70 | 0.482 |
|  |  | Conduct | 0.0038 | 0.55 | 0.582 |
| Cognition  Bonferroni Corrected p value threshold: p <.007 | | Flanker | 0.0181 | 2.38 | 0.017 |
|  |  | Pattern Comparison | -0.0078 | -1.09 | 0.278 |
|  |  | Picture Sequence | 0.0134 | 1.82 | 0.069 |
|  |  | List Sorting | -0.0085 | -0.87 | 0.386 |
|  |  | Card Sort | -0.0185 | -1.86 | 0.063 |
|  |  | Picture Vocab | 0.0289 | 4.53 | <.001* |
|  |  | Oral Reading | 0.0332 | 5.19 | <.001* |

Results of 13 linear mixed effects models with mental health and cognitive domains as the independent variables. Each including PNS as the dependent variable and controlling for BIS, objective neighborhood violence, age, sex, family income, area deprivation index, and population density. Each model was subset by family and research site. The top table contains the estimates for PNS, and the bottom contains the estimates for BIS.

**Supplementary Table 23: Relationship of Perceived Neighborhood Safety to Cognition and Mental Health Outcomes, When Controlling for Self-Reported Direct Violence Exposure**

|  |  | Outcome | beta estimate | t value | | p value |
| --- | --- | --- | --- | --- | --- | --- |
|  |  | **Perceived Neighborhood Safety (controlling for Individual)** | | | | |
| Mental Health  Bonferroni Corrected p value threshold: p <.008 | | Depression | -0.0846 | -5.96 | <.001* | |
|  |  | Anxiety | -0.0559 | -4.29 | <.001* | |
|  |  | Somatic | -0.0688 | -5.21 | <.001* | |
|  |  | ADHD | -0.0989 | -7.75 | <.001* | |
|  |  | Oppositional Defiant | -0.0573 | -4.40 | <.001* | |
|  |  | Conduct | -0.0527 | -4.05 | <.001* | |
| Cognition  Bonferroni Corrected p value threshold: p <.007 | | Flanker | 0.0471 | 3.49 | <.001* | |
|  |  | Pattern Comparison | 0.0606 | 5.02 | <.001* | |
|  |  | Picture Sequence | 0.0590 | 4.65 | <.001* | |
|  |  | List Sorting |  |  |  | |
|  |  | Card Sort |  |  |  | |
|  |  | Picture Vocab | 0.0211 | 1.86 | 0.063 | |
|  |  | Oral Reading | 0.0249 | 2.17 | 0.030 | |
|  |  | **Individual-level Violence (controlling for PNS)** | | | | |
| Mental Health Bonferroni Corrected p value threshold: p <.008 | | Depression | 0.0292 | 2.23 | 0.026 | |
|  |  | Anxiety | 0.0227 | 1.89 | 0.060 | |
|  |  | Somatic | 0.0276 | 2.27 | 0.024 | |
|  |  | ADHD | 0.0575 | 4.89 | <.001* | |
|  |  | Oppositional Defiant | 0.0707 | 5.89 | <.001* | |
|  |  | Conduct | 0.0805 | 6.70 | <.001* | |
| Cognition  Bonferroni Corrected p value threshold: p <.007 | | Flanker | -0.0052 | -0.42 | 0.675 | |
|  |  | Pattern Comparison | -0.0172 | -1.54 | 0.124 | |
|  |  | Picture Sequence | -0.0299 | -2.56 | 0.011 | |
|  |  | List Sorting |  |  |  | |
|  |  | Card Sort |  |  |  | |
|  |  | Picture Vocab | -0.0193 | -1.85 | 0.064 | |
|  |  | Oral Reading | -0.0062 | -0.59 | 0.554 | |

Results of 13 linear mixed effects models including mental health and cognition as dependent variables and PNS as the independent variable, controlling for individual-level violence exposure. Each model included sex, age, objective neighborhood violence, ADI, and family income as covariates, subset by family and research site. The upper part of the table displays the estimate associated with PNS and the lower portion estimates associated with individual-level violence. Results are not displayed for list sorting or card sorting because both were only collected at baseline and individual-level violence exposure was only available at year 2.
